# Ergot in Causing Retention of Placenta

**Published:** 1854-01

**Authors:** Charles Hasbrouck

**Affiliations:** Rockland Co., N. Y.


### ECLECTIC DEPARTMENT.

---

### *Action of Ergot in causing Retention of the Placenta.*

BY CHARLES HASBROUCK, M. D., ROCKLAND CO., N. Y.

"Spasmodic contraction," or "premature contraction" of the cervix uteri, is referred to by obstetrical writers as an occasional cause of retention of the placenta; and Dewees, in his *System of Midwifery*, mentions the fact, that in some instances of this character, the body of the uterus is also found to be "hard and well contracted." But so far as my information extends, the frequent relation between this condition of the uterus and the administration of ergot during labor has never been pointed out. The following cases, it seems to me, are calculated to show that such relation does occasionally exist; that the placenta may, perhaps not unfrequently, be retained by the permanent and uniform contraction of every part of the uterus, excited by the specific action of egot upon the organ.

Case 1.—Mrs. P—— D——, aged about twenty years, was taken in labor with her first child, Sept. 20th, 1842. He labor progressed slowly with pains of moderate severity for about fifteen hours, when, without any appreciable cause, they entirely ceased. The vertex, at this time, was found to occupy the superior strait with the occiput to the left acetabulum. The uterus remaining inactive, and the soft parts being perfectly relaxed, I ruptured the membranes, in the hope of reviving uterine contraction. No pains ensuing, however, and nearly twenty-four hours having elapsed since the commencement of her labor, I emptied the bladder and rectum, and administered, in divided doses, a drachm of the egot in decoction. The peculiar action of the drug soon manifested itself in the powerful and almost constant contractions which ensued, and in about an hour the child was expelled, completely asphyxiated. On placing my hand upon the hypogastrium, I was astonished at the unusual hardness of uterine globe. But although the body of the uterus was thus firmly contracted, the woman was entirely free from pain. On following up the cord, the contraction was found to extend to the cervix and os uteri. The placenta was beyond the reach of my finger. I waited an hour and a half, hoping that the cervix uteri would relax sufficiently to allow the placenta to pass; but the whole uterus remaining firmly contracted, I introduced my hand into the vagina, and with great difficulty succeded in dilating the os sufficiently to enable me to grasp, or rather to hook down the placenta. The patient convalesced slowly with symptoms of prolapsus uteri.

CASE II.—Mrs. J——— B———, aged nineteen years, was taken in labor with her first child, Sept. 14th, 1843. From the commencement of labor, her pains were short, infrequent, and inefficient. She had been in labor twenty-four hours, her pains having become expulsive, though still short and inefficient, the vertex being engaged in the superior strait, the os uteri being fully dilated, and the membranes protruding almost to the os externum, when she was suddenly seized with violent convulsions, which continued to recur every half hour or hour during the subsequent progress of her labor. After copious venesection, &c., I emptied the bladder, and gave a decoction of a drachm of ergot, in divided doses, without any appreciable effect in hurrying the action of the uterus. The vertex, however, continued to advance, and had got low down in the pelvis, when, upon the suggestion of Dr. R. W. Stevenson, who now saw the patient with me, a tablespoonful of very strong decoction of the ergot was given every fifteen minutes. The uterus gradually responded to the stimulus of the ergot, and the child was slowly expelled, twenty-four hours after the commencement of labor. The woman now lay perfectly unconscious, while the convulsions continued to recur. The uterus was fully contracted, presented the same unusually hard and solid feel as in the above case, while the placenta was firmly retained by the contracted state of its cervix and os. After waiting two hours without any change occurring in the condition of the uterus, I passed my hand into the vagina, and succeeded with considerable effort, in dilating the os uteri sufficiently to enable me to reach the placenta, which, together with my hand, was immediately expelled. The woman had a tedious but safe convalescence.

CASE III.—Mrs. M——— D———, aged 39 years, was taken in labor with her first child, Sept. 2nd, 1848, forty-eight hours after the spontaneous rupture of the membranes. Her labor progressed, but regularly, for thirty-six hours. The os uteri being then dilated, the soft parts relaxed, and the vertex occupying the superior strait, while the powers of the uterus seemed to flag, her pains becoming shorter and less frequent, I gave her the ergot without producing any effect. Four hour later, the patient having procured some sleep from a dose of morphine, I gave her more of a very strong decoction of ergot, regardless of quantity. The peculiar pains of ergotism soon followed, the vertex advanced, and the child was shortly delivered. The uterus immediately contracted upon the placenta, feeling like marble under my hand. Neither pain nor flooding was present, and on following up the chord, the os uteri was also found contracted to about the size of a quarter of a dollar, presenting a firm, almost cartilaginous feel.

I waited an hour and a half, hoping that the uterus would relax, but fearing that any longer delay would only tend to increase the difficulty of overcoming the stricture (if I may so term it) of the os uteri, I introduced my hand into the vagina, but found it impossible, without using an unwarrantable degree of violence, than to pass more than a couple of fingers into the os uteri. With these, I succeeded after a considerable effort in hooking down the enclosed placenta. The patient had a rapid covalescence.

Case IV.—Mrs. A——— B———, aged 20 years, was confined with her first child, Oct. 22d, 1850. From the commencement of her labor, the uterus acted feebly, the pains being extremely short and infrequent. Her labor progressed slowly however, and in twelve hours the os uteri was fully dilated. Her pains now became expulsive, but the vertex advanced with extreme slowness, in consequence of the shortness of each pain. Hoping to excite the uterus to more vigorous action, I ruptured the membranes; but failing in this, and the vertex occupying the pelvic cavity, fifteen hours after the commencement of labor, and three hours after the discharge of the liquor amnii, I gave the decoction of a drachm of ergot. This had the desired effect, and the woman was soon delivered of a fine, healthy girl. On placing my hand upon the hypogastrium, the uterus was found to be hard and well contracted. As soon as the child was separated from the mother, I passed my finger along the cord and found the placenta lying over the os uteri, which was also contracted to the size of a half a dollar, and moderately firm. Feeling confident that it would be useless to wait for the spontaneous expulsion of the placenta, and believing that delay would only increase the difficulty of its manual delivery, by giving the crevix and os uteri more time to become more permanently contracted, I at once passed my hand into the vagina, and with but little difficulty succeeded in dilating the os, so as to reach and withdraw the placenta. The woman had a good "getting up."

It is well known that the placenta is ordinarily detached and expelled, a few minutes after the birth of the child, with little or none of the peculiar pain that attends the parturient contraction of the womb. Indeed, it appears that the uniform contraction of the whole uterus, the general shortening of all the uterine fibres, the circular, as well as longitudinal, which necessarily follows the expulsion of so large a portion of the contents of the uterus, in other words, the simple tonic contraction of that organ, is all that is usually necessary to accomplish this result. Hence arises the rule of practice, when the placenta is retained in consequence of uterine inertia, that by frictions, cold, ergot, &c., we must endeavor to excite the tonic contraction of the womb.

But in this, as in other matters, extremes occasionally meet, and the same action of the uterus which, when moderate and normal, will usually accomplish the delivery of the placenta, becomes when abnormal and excessive, the very means of retarding its expulsion, and of rendering its delivery very difficult. And such was the difficulty in the above cases. The uterus was not felt high up, nor flabby, as in uterine inertia, but was found low down in the abdomen, and presented to the feel that marble hardness which is both the evidence and result of strong uterine contraction. Not the least flooding was present. Indeed, there was no discharge of any kind whatever; and I also noticed an entire abscence of that alternate relaxation and contraction of the womb which usually follow the expulsion of the child, and which can easily be felt by the hand placed upon the woman's abdomen. In short, the placenta was completely encysted, and firmly retained, in consequence of the permanent tonic contraction of the uterus being prematurely and excessively developed; and that this premature and excessive contraction resulted from the specific action of the ergot upon uterus, there can scarcely be a doubt.

The action of ergot seems to be principally directed to the lower portion of the spinal cord, promoting in an extraordinary degree the innervation and tone of the muscles and tissues to which the lumbar and sacral nerves, and the nerves of the hypogastric plexus are disturbed. This is proved by the benefit that results from its use in paralysis of the bladder, and in partial or incomplete paraplegia; and it is probably by virtue of the same action that it is of such essential service, as I have frequently observed, in the sciatic pains and weakness of relaxed and debilitated habits.

The uterus, it is true, is supplied with nerves from the sympathetic system, and most probably "is not itself dependent upon the spinal cord for power of contraction." (*Carpenter.*) This would seem probable from the fact, that delivery has taken place by the spontaneous contractions of the uterus, in cases of parpalegia from disease of the lower part of the spinal cord. But while the peristaltic or parturient contractions of the uterus, like the contractions of the heart, or intensities, may perhaps be, in a great measure, independent of its nervous connection with the spine, yet its numerous reflex sympathies and actions prove conclusively that the several nerves of the cerebrospinal system which pass into the plexuses of the sympathetic, and are thus distributed to the uterus, are not without their appropriate influence. Indeed, it would seem probable, that while the peristaltic action of the uterus, as in ordinary parturition, is in some way connected with its supply of ganglionic nerves, its condition of muscular tonicity, or its tonic contraction, may depend upon its connection with the spine. And as irritation of a motor nerve excites contraction in every part of the muscle supplied by that nerve, so the powerful influence exerted by the ergot upon that part of the spinal cord from which the spinal branches of the uterine nerves are derived, excites every part of the uterus, its cervix and os, as well as its fundus and body, to strong and permanent contraction, thus offering, as in the above cases, a very serious impediment to the passage of the placental mass.

I am not sure that these suggestions harmonize with received opinions in relation to the physiology of uterine contraction. But whether they do or do not, or whatever may be the *modus operandi* of the ergot, there can be no uncertainty in relation to the fact, that it possesses the property of increasing the tone, and promoting the contraction of the uterine fibre. Its beneficial effects in passive menorrhagia, that protracted and exhausting drain from the uterine vessels so often met with in relaxed and leucorrhœal habits, prove that it is capable of increasing the innervation and organic contractility of the capillaries of the womb ; and no one engaged in obstetrical practice can doubt its power of exciting the muscles of that organ to prompt and powerful contraction, when given in the progress of labor.

The contractions thus excited differ, however from the ordinary contractions of parturiton, in partaking less of a peristaltic character, being more constant, and affecting equally the circular and longitudinal fibres of every part of the uterus ; and it can be easily understood that the cervix and os uteri may thus become so firmly contracted under the influence of large doses of ergot, as to offer a complete obstacle to the delivery of the placenta. Indeed, so entirely convinced have I become of this danger, from the results of my own observation that laterly, whenever I can find it necessary to administer egot to expedite the expulsion of the child, (and particularly in first labors, in which the tonic contraction of the uterus is generally more promptly and more perfectly established,) I have at once proceeded to the delivery of the placenta, ass soon as possible after the separation of the child. By acting thus early, as soon as the tonic contraction of the womb is decidedly developed, but before it becomes permently and completely established, I have, I think, in several instances, avoided the difficulty which was encountered in the above cases.—*N. Y. Jour. Medicine.*

